# Clinical characteristics, diagnostic modalities, and therapeutic strategies of spontaneous renal artery dissection: A systematic review and diagnostic analysis

**DOI:** 10.1371/journal.pone.0340766

**Published:** 2026-01-23

**Authors:** Jiahao Zhu, Zengqiang Xu, Yang Geng, Mengxin Jiang, Chang Xu, Yingjiang Xu

**Affiliations:** 1 Binzhou Medical University Hospital, 661 Huanghe Rd, Binzhou, P.R. China; 2 School of Medical Imaging (Binzhou Medical University), 522 Huanghe Rd, Binzhou, P.R. China; 3 The First School of Clinical Medicine (Binzhou Medical University), 522 Huanghe Rd, Binzhou, P.R. China; Azienda Ospedaliero Universitaria Careggi, ITALY

## Abstract

**Objective:**

To investigate the clinical features, diagnostic approaches, and treatment strategies of spontaneous renal artery dissection (SRAD) through systematic review and data analysis, thereby providing evidence-based insights for optimizing clinical management.

**Methods:**

A systematic search was conducted across PubMed, Embase, Cochrane Library, Web of Science, China National Knowledge Infrastructure (CNKI), and Wanfang Database. Case-control studies, cohort studies, and case series were included. Demographic data, symptomatology, diagnostic modalities, treatment regimens, and clinical outcomes were extracted. Statistical analyses were performed using RStudio.

**Results:**

A total of 73 case reports involving 97 patients were included. The mean patient age was 46 years, with a male predominance (82.5%, 80/97). The most common presenting symptom was acute-onset flank pain (74.2%), and comorbid hypertension was observed in 61.9% of cases. Computed tomography angiography (CTA) was the primary diagnostic modality (85.6%), with multimodal imaging utilized in 87.6% of cases. Revascularization rates differed significantly between conservative management (37.1%), endovascular intervention (71.0%), and surgical intervention (6.2%) (P < 0.01). Regarding blood pressure outcomes: the proportion of patients achieving normotension without medication was significantly higher in the non-conservative group (54.1%, 20/37) than in the conservative group (30.0%, 6/20) (P < 0.05); however, there was no statistically significant disparity in the overall blood pressure control rate (normotension without medication + controlled with medication) between groups (P > 0.05). Overall mortality was 3.1%, and renal function deterioration occurred in 30.9% of patients.

**Conclusion:**

Management of SRAD necessitates individualized decision-making. Conservative therapy remains appropriate for most patients, while endovascular intervention demonstrates superior revascularization efficacy in cases with severe symptomatology or dissection progression. Prospective studies are warranted to validate therapeutic disparities and establish standardized diagnostic and treatment protocols.

## 1. Introduction

Spontaneous renal artery dissection (SRAD) is a rare non-traumatic vascular disorder (annual incidence: 0.5–1.5 per 100,000; 1%–2% of renal artery pathologies) characterized by spontaneous renal artery intimal tear, false lumen formation, and potential complications including renal ischemia, infarction, or irreversible impairment [1,2]. Key pathogenic factors include arterial medial degeneration, hypertension, fibromuscular dysplasia (FMD), and hereditary vasculopathies [1,3]. Its clinically insidious presentation and overlap with common acute abdominal conditions (e.g., nephrolithiasis, pyelonephritis) lead to underdiagnosis rates exceeding 30%, even with advancements in non-invasive imaging (CTA, MRA) [4,5].

Clinical management of SRAD is hindered by two critical gaps: (1) reliance on small-sample case reports/retrospective studies (no large-scale RCTs or cohort studies) leading to controversy over optimal therapies (conservative, endovascular, open surgery); and (2) heterogeneous outcome assessment criteria (e.g., blood pressure normalization, revascularization success) that impede unified efficacy evaluation [5]. Discrepancies in study findings—e.g., conflicting support for early endovascular intervention vs. conservative management in stable patients—undermine evidence-based decision-making and standardized guideline development.

To address these gaps, this study conducted a systematic review of global SRAD case reports and cohort studies published between 1991 and 2024, pooling clinical data from 97 patients. The objectives are threefold: (1) to comprehensively delineate the epidemiological profile, clinical manifestations, and imaging-based diagnostic patterns of SRAD; (2) to compare the efficacy of diverse therapeutic strategies (conservative, endovascular, surgical) in achieving revascularization, blood pressure control, and renal function preservation; and (3) to evaluate complication rates and prognostic risks, thereby generating evidence-based recommendations for individualized management. The findings aim to address current evidence gaps, facilitating a paradigm shift from empirical to evidence-based management of SRAD and ultimately improving long-term patient outcomes.

## 2. Materials and methods

Ethical approval for this study was provided by the Ethics Committee of the Affiliated Hospital of Binzhou Medical College.

### 2.1. Literature search and screening

This systematic review adhered to the Preferred Reporting Items for Systematic Reviews guidelines.

**Fig 1:**
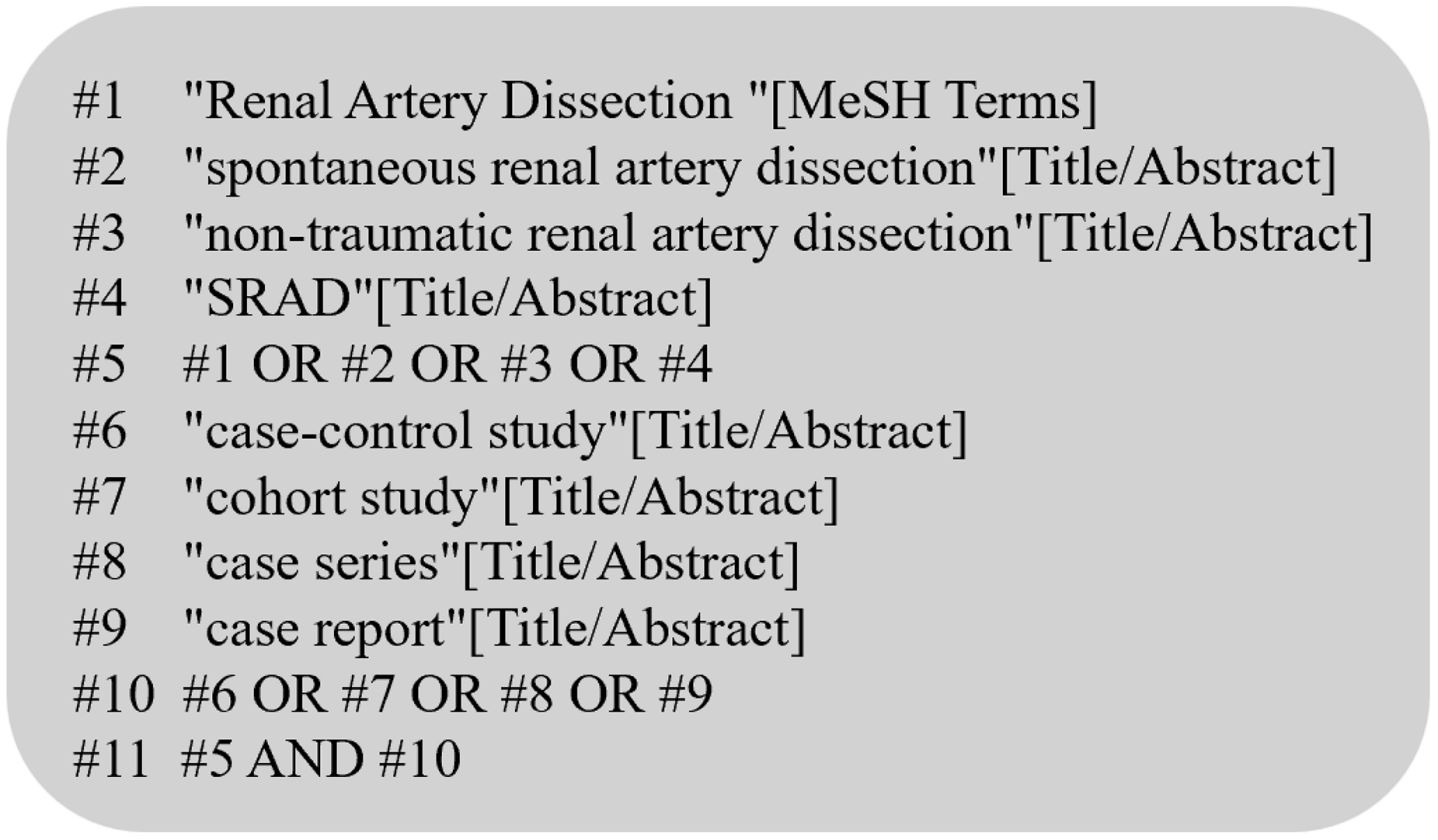
Searching strategies in PubMed.

A comprehensive search was conducted in PubMed, Embase, Cochrane Library, Web of Science, China National Knowledge Infrastructure (CNKI), and Wanfang Database for case-control studies, cohort studies, and case series on spontaneous renal artery dissection (SRAD), with the search period spanning from database inception to December 2024. English search terms included: *spontaneous renal artery dissection*, *SRAD*, *non-traumatic renal artery dissection*, *diagnosis*, *treatment*, *prognosis*, and *outcomes*. Chinese search terms comprised: zi fa xing shen dong mai jia ceng, fei chuang shang xingshen dong mai jia ceng, zhen duan and yu hou. The search strategy combined Medical Subject Headings (MeSH) terms with free-text keywords. Manual screening of reference lists from included studies and authoritative journals in nephrology (e.g., *Journal of Vascular Surgery*, *Nephrology*) was performed to ensure comprehensiveness. Two investigators (JZ and ZX) independently conducted the manual search to verify completeness and accuracy. The PubMed search strategy is detailed in Fig 1.

### 2.2. Inclusion and exclusion criteria

#### 2.2.1. Study types.

Case-control studies, cohort studies, and case series were included. Conference abstracts and studies lacking control groups (except for prognostic analyses) were excluded.

#### 2.2.2. Interventions.

Studies comparing diagnostic accuracy across imaging modalities (e.g., CTA, MRA, DSA) or therapeutic efficacy between conservative management (anticoagulation/antihypertensive therapy), endovascular intervention (stent placement/angioplasty), and open surgery were included.

#### 2.2.3. Participants.

Patients with SRAD confirmed by imaging (CTA/MRA/DSA) or histopathology, without a history of trauma or iatrogenic causes.

#### 2.2.4. Outcome measures.

Blood pressure recovery: Normalized (without medication), partially controlled (requiring medication), or uncontrolled.

Revascularization success or failure.

Renal function: Stabilized (serum creatinine fluctuation <20%), improved (serum creatinine reduction >20%), or deteriorated (serum creatinine elevation >20%).

Survival: Alive (follow-up ≥3 months) or deceased.

#### 2.2.5. Exclusion criteria.

A. Reviews or conference abstracts; B. Duplicate publications; C. Animal experiments or cellular studies; D. Studies with duplicated data or insufficient extractable key information; E. Cases of renal artery dissection secondary to vascular interventions, trauma, or extensive aortic/visceral/mesenteric artery dissections.

### 2.3. Literature screening and data extraction

Two investigators independently extracted the following data:

a Study characteristics: Title, first author, study design, journal, publication year.
b Baseline patient data: Sample size, demographics (age, sex, comorbidities), symptoms (flank pain, hypertension, renal dysfunction).
c Key risk-of-bias elements.
d Outcomes: Diagnostic modalities (CTA/MRA/DSA), treatment strategies (conservative, endovascular, surgical repair), and outcomes (revascularization, therapeutic efficacy, complications, follow-up). Discrepancies were resolved through consensus discussions.

### 2.4. Quality appraisal and risk of bias assessment

The methodological quality of the 73 included studies was evaluated using the CARE (Case Report) guidelines and Joanna Briggs Institute (JBI) Critical Appraisal Checklist for Case Reports. Two independent reviewers conducted the quality assessment, with disagreements resolved via consensus.

### 2.5. Statistical analysis

Data were analyzed using RStudio. Categorical variables were analyzed using chi-square tests and reported as percentages with 95% confidence intervals (CI). A two-tailed *P*-value ≤0.05 was considered statistically significant.

## 3. Results

### 3.1. Literature search results

**Fig 2:**
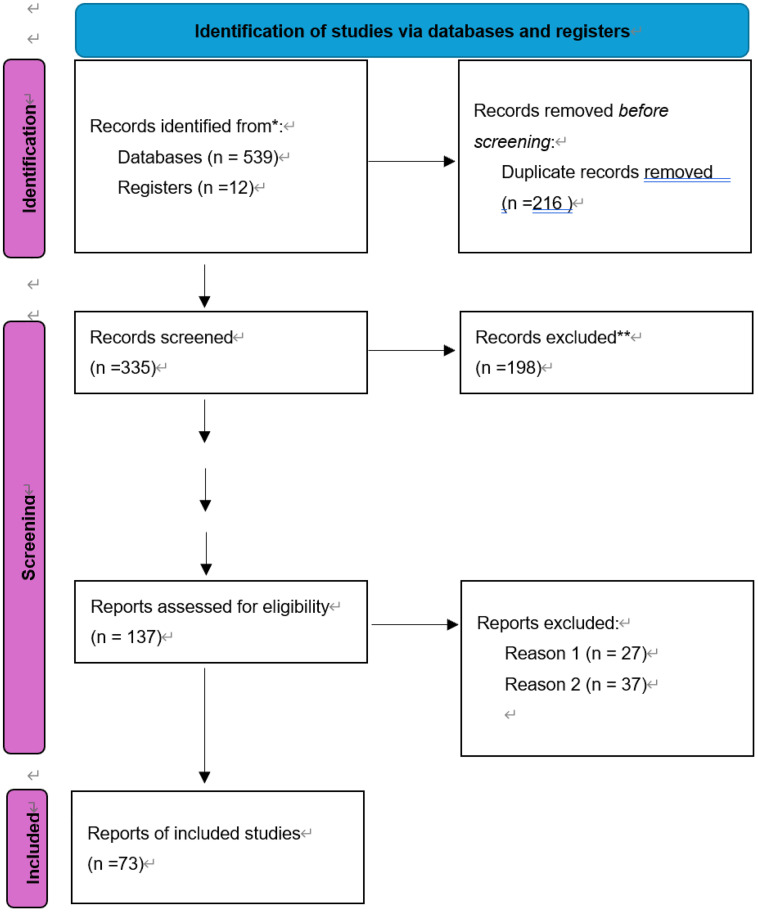
Literature Screening Process and Results *The databases searched and the number of documents detected are specified as follows: PubMed (n = 141), EMbase (n = 4), The Cochrane Library (n = 43), WOS (n = 163), CNKI (n = 138), WanFang Date (n = 50).

The initial search yielded 551 potentially relevant articles, including 141 from PubMed, 43 from Cochrane Library, 4 from Embase, 163 from Web of Science, 138 from China National Knowledge Infrastructure (CNKI), and 50 from Wanfang Database. After deduplication, 335 articles underwent title and abstract screening, with 198 excluded due to insufficient data or mismatched study types. Full-text review of 137 articles led to the inclusion of 73 case reports involving 97 patients with spontaneous renal artery dissection (SRAD). The screening flowchart is illustrated in Fig 2.

### 3.2. Quality appraisal and risk of bias assessment

Methodological quality of the 73 studies was assessed using the CARE guidelines and Joanna Briggs Institute (JBI) Critical Appraisal Checklist for Case Reports. A summary table of quality evaluation results and risk assessment of bias is shown in Table 1. Two independent reviewers achieved substantial agreement (Kappa = 0.72), with discrepancies resolved via consensus.

**Table 1:** Quality evaluation results and risk of bias assessment.

Number	CARE Score (/13)	JBI Score (/8)	Customized Score (/3)	Total Quality Grade	Selection Bias	Information Bias	Confounding Bias	Consolidated Risk Of Bias
1	10.5	7	3	High	Low	Low	Medium	Low
2	11	7	3	High	Low	Low	Low	Low
3	9.5	6	2 (ESWL effects not excluded)	Medium	Medium	Medium	Medium	Medium
4	8	5	3	Medium	Low	Medium	Low	Medium
5	10	6	3	High	Low	Low	Medium	Low
6	7.5	5	2 (not mentioned in follow-up)	Medium	Medium	High	Medium	Medium
7	11	7	3	High	Low	Low	Low	Low
8	9	6	2 (multivessel mixing)	Medium	Medium	Medium	High	Medium
9	8.5	5	3	Medium	Low	Medium	Low	Medium
10	9	6	2 (nephrectomy not analyzed)	Medium	Medium	Medium	Medium	Medium
11	7	4	2 (missing data)	Low	High	Medium	Medium	Medium
12	10	6	3	High	Low	Low	Low	Low
13	8.5	5	2 (COPD not analyzed)	Medium	Medium	Medium	High	Medium
14	7	4	2 (absence of underlying disease)	Low	Medium	High	Medium	Medium
15	9.5	6	3	High	Low	Low	Low	Low
16	8	5	2 (SMA mixed)	Medium	Medium	Medium	High	Medium
17	6.5	3	1 (multiple cases combined)	Low	High	High	Medium	High
18	8	5	3	Medium	Low	Medium	Low	Medium
19	7.5	4	2 (FMD not validated)	Medium	Medium	Medium	Medium	Medium
20	7	4	1 (Bone fragments not ruled out)	Low	High	High	Medium	High
21	8	5	2 (insufficient pathological evidence)	Medium	Medium	Medium	Medium	Medium
22	6	3	1 (ambiguous ending)	Low	High	High	High	High
23	9	6	3	High	Low	Low	Low	Low
24	8.5	5	2 (diverticulosis mixed)	Medium	Medium	Medium	High	Medium
25	7	4	2 (genetic diseases not analyzed)	Low	Medium	High	High	High
26	8	5	3	Medium	Low	Medium	Low	Medium
27	7.5	4	2 (uncontrolled hepatitis)	Medium	Medium	Medium	High	Medium
28	6.5	3	1 (follow-up only 17 days)	Low	High	High	Medium	High
29	5	2	0 (no follow-up)	Low	High	High	High	High
30	9	6	3	High	Low	Low	Low	Low
31	4	2	0 (incomplete data on deaths)	Low	High	High	High	High
32	7	4	2 (spontaneous recirculation not verified)	Low	Medium	Medium	Medium	Medium
33	8	5	3	Medium	Low	Medium	Low	Medium
34	7.5	4	2 (restenosis not analyzed)	Medium	Medium	High	Medium	Medium
35	6	3	1 (short-term follow-up)	Low	High	High	Medium	High
36	7	4	2 (COVID mixing)	Low	Medium	Medium	High	Medium
37	8	5	3	Medium	Low	Medium	Low	Medium
38	6.5	3	1 (multiple cases combined)	Low	High	High	Medium	High
39	7	4	2 (ischemic mechanism ambiguous)	Low	Medium	Medium	Medium	Medium
40	6	3	1 (missing data)	Low	High	High	High	High
41	7.5	4	2 (mixed not analyzed)	Medium	Medium	Medium	High	Medium
42	8	5	3	Medium	Low	Medium	Low	Medium
43	9	6	3	High	Low	Low	Low	Low
44	7	4	2 (nephrectomy not analyzed)	Low	Medium	Medium	Medium	Medium
45	8	5	2 (mixed aortic coarctation)	Medium	Medium	Medium	High	Medium
46	7.5	4	2 (misdiagnosis not specified)	Medium	Medium	Medium	Medium	Medium
47	6	3	1 (unanticoagulated)	Low	High	High	Medium	High
48	8	5	3	Medium	Low	Medium	Low	Medium
49	5	2	0 (hemolytic misdiagnosis)	Low	High	High	High	High
50	7	4	2 (misdiagnosis not analyzed)	Low	Medium	Medium	Medium	Medium
51	8	5	3	Medium	Low	Medium	Low	Medium
52	7	4	2 (cannabis not analyzed)	Low	Medium	Medium	High	Medium
53	7.5	4	2 (thrombus not analyzed)	Medium	Medium	Medium	Medium	Medium
54	6	3	1 (emergency not validated)	Low	High	High	Medium	High
55	7	4	2 (misdiagnosis not ruled out)	Low	Medium	Medium	Medium	Medium
56	4	1	0 (death data missing)	Low	High	High	High	High
57	5	2	0 (autopsy not analyzed)	Low	High	High	High	High
58	6	3	1 (thrombolysis not verified)	Low	Medium	High	Medium	High
59	7	4	2 (movement mixing)	Low	Medium	Medium	Medium	Medium
60	5	2	0 (multiple cases combined)	Low	High	High	High	High
61	7	4	2 (conservative treatment not analyzed)	Low	Medium	Medium	Medium	Medium
62	6	3	1 (stent failure)	Low	High	High	Medium	High
63	7	4	2 (hypothermia not analyzed)	Low	Medium	Medium	Medium	Medium
64	8	5	3	Medium	Low	Medium	Low	Medium
65	6.5	3	1 (partial renal impairment)	Low	Medium	High	Medium	High
66	7	4	2 (short-term follow-up)	Low	Medium	Medium	Medium	Medium
67	9	6	3	High	Low	Low	Low	Low
68	7	4	2 (thrombolysis not verified)	Low	Medium	Medium	Medium	Medium
69	8	5	2 (mixed history of trauma)	Medium	Medium	Medium	High	Medium
70	9	6	3	High	Low	Low	Low	Low
71	7	4	2 (non-operated)	Low	Medium	Medium	Medium	Medium
72	5	2	0 (missing data)	Low	High	High	High	High
73	8	5	2 (mixed atherosclerosis)	Medium	Medium	Medium	High	Medium

Quality tiers were defined as follows

High quality (CARE score ≥10 + JBI score ≥6 + custom criteria ≥2): 18 studies (24.7%), characterized by robust imaging evidence (CTA + DSA), follow-up ≥6 months, and detailed therapeutic protocols.

Moderate quality (CARE 7–9 + JBI 4–5 + custom criteria ≥1): 31 studies (42.5%), with limitations including incomplete data (e.g., smoking history omission) and short follow-up (3 months).

Low quality (CARE ≤6 + JBI ≤ 3 + custom criteria ≤1): 24 studies (32.8%), marked by failure to exclude secondary causes, reliance on single imaging modality (e.g., ultrasound only), and vague outcome descriptions.

Risk of bias was stratified as

Low risk (22 studies, 30.1%): Adequate control of selection bias (exclusion of secondary causes), information bias (gold-standard imaging + complete follow-up), and confounding factors (e.g., comorbidity adjustment).

Moderate risk (35 studies, 47.9%): Partial adherence to quality criteria without critical flaws.

High risk (16 studies, 21.9%): Major deficiencies, including key data gaps (e.g., survival bias) or reliance on single diagnostic modality.

Key limitations included short-term follow-up (28.8%), inadequate confounding control (41.1%), and non-multimodal imaging (19.2%).

### 3.3. Baseline characteristics of included studies

Studies spanned 1991–2024 (Table 2, Table 3). The cohort comprised 97 patients (mean age 47.6 ± 11.3 years; median 46 years), with male predominance (82.5%, 80/97). Hypertension was present in 61.9% (60/97) at onset, with 23 cases reporting specific blood pressure values (mean systolic 167 ± 24 mmHg, diastolic 98 ± 18 mmHg). Smoking history was noted in 18.6% (18/97). Comorbidities included preexisting hypertension (11.3%, 11/97), fibromuscular dysplasia (FMD, 16.5%, 16/97), diabetes mellitus (2.1%, 2/97), and cardiovascular disease (e.g., coronary artery disease, 7.2%, 7/97). Presenting symptoms included acute-onset flank pain (74.2%, 72/97), hematuria (13.4%, 13/97), and hypertensive crisis (16.5%, 16/97). Diagnostic delay occurred in 8.2% (8/97), with misdiagnoses as nephrolithiasis (3 cases), pyelonephritis (6 cases), or renal vein thrombosis (1 case).

**Table 2:** Clinical characteristics of patients included in the literature.

Author	Year	Sex	Age	Onset with hypertension	Initial diagnosis	History of smoking	Main point (of an argument)	Recovery of blood pressure	Revascularization
James W.H. Macneil	2017	Male	69	Yes(158/94 mmHg)	Acute kidney injury, right kidney infarction	Not mentioned	Right-sided dystocia, nausea, vomiting	Control (drugs)	Yes
Guangqing Yuan	2023	Male	51	Yes	Right renal artery entrapment with partial renal infarction	Not mentioned	Pain in the right abdomen and lower back	Normal	Yes
Ozbek Orhan	2011	Male	37	Yes(200/110 mmHg)	Bilateral renal artery entrapment (possibly related to ESWL)	Yes	Bilateral dystocia, hematuria, headache	Control (drugs)	Yes
Mustafa Korkut	2020	Male	25	No (normal on admission)	entrapment of the left renal artery	Yes	Left dystocia (worse with exercise)	Normal	Yes
F Del Porto	2020	Male	46	Yes	Bilateral renal artery entrapment + positive antiphospholipid antibodies	Yes	Sudden right upper abdominal pain, diarrhea	Control (drugs)	Yes
Luana Gatto	2020	Male	37	Yes	Fibromuscular dysplasia (FMD) with vertebral artery entrapment aneurysm	Not mentioned	Sudden onset headache, vomiting (SAH)	Control (drugs)	Yes
Gerardo A. Vitiello	2017	Male	40	Yes(152/102 mmHg)	Left renal artery entrapment with renal infarction	Not mentioned	Recurrence of left-sided dystocia	Normal	Yes
Tadahisa Sugiura	2011	Male	30	Yes (hypertension on admission)	Fibromuscular dysplasia (FMD) with four peripheral arterial entrapments	Not mentioned	Sudden severe abdominal pain, headache, dizziness	Control (drugs)	Yes
M Alonso-Alcañiz	2017	Male	45	Yes (148/96 mmHg)	Left renal artery entrapment with partial renal infarction	Yes (20/day)	Left dystocia (motion-independent)	Control (drugs)	Yes
Jing Lei	2022	Female	45	Yes (220/120 mmHg)	Right renal artery entrapment with renal infarction	Not mentioned	Right-sided dystocia, abdominal pain	Normal	Yes (Nephrectomy)
Naman S. Desai	2013	Famle	65	Yes (144/78 mmHg)	Bilateral renal artery entrapment with renal infarction	Not mentioned	Left dystocia, hematuria	Control (drugs)	Yes
Bora Peynircioglu	2011	Male	52	Yes (170/100 mmHg)	Right renal artery entrapment with renal vascular hypertension	Not mentioned	hypertension out of control	Normal	Yes
Gaurav Tandon	2012	Male	65	Yes (240/150 mmHg)	right renal artery entrapment	Yes (30/year)	Hypertensive crisis, depressive symptoms	Normal	Yes
Ainhoa García	2014	Male	59	Yes (195/113 mmHg)	Left renal artery entrapment with renal infarction	Yes	Left-sided dystocia, hypertension	Normal	Yes
Gaetano Ferrara	2024	Male	49	Yes (160/90 mmHg)	Bilateral renal artery entrapment with multiple renal infarcts	Not mentioned	Bilateral dystocia, headache, fatigue	Control (drugs)	No
Naiding Zhang	2021	Male	48	No	Abdominal pain, diarrhea, bloating	No	Sudden abdominal and back pain	Normal	Yes
Sonia Ramamoorthy	2002	Male3Famle1	51	2Yes2No	Abdominal pain, fever, hematuria	Not mentioned	Abdominal pain, fever, hematuria	Normal	No
Sang Hak Lee	2003	Male	48	No	Suspected appendicitis or kidney stones	No	Right abdominal and lumbar pain	Normal	Yes
Amir Alamir	1997	Male	47	No	Suspected kidney stones	No	sudden onset of low back pain	Normal	No
Mehmet Beyazal	2023	Famle	65	No	Right abdominal pain, nausea and vomiting	Not mentioned	Sharp pain in the right abdomen	Normal	No
Nitin Kolhe	2004	Male	42	No	Low back pain, microscopic hematuria	Not mentioned	Low back pain, hematuria	Normal	No
Susannah Boulet	2023	Male	51	Yes (history of hypertension)	Suspected abdominal injury	Yes	Left lower back pain	Control (drugs)	No
Yasutoshi Yoshiyama	2021	Male	53	No	lumbago	Yes	sudden onset of low back pain	Normal	Yes
Stefano Bonardelli	2013	Male	54	Yes (history of hypertension)	Chest and back pain, abdominal pain	Yes	Chest and back pain, abdominal pain	Control (drugs)	Yes
Richard Conway	2012	Male	25	No	Right lower back pain	No	Sudden right lower back pain	Normal	No
Chin-Ming Su	2004	Male	41	Yes (180/100 mmHg)	renal artery infarction	Not mentioned	Sudden right lower back pain	Normal	Yes
Marcio Miyamoto	2018	Male	40	Yes (not controlled)	secondary hypertension	Yes	Recent exacerbation of hypertension	Control (drugs)	Yes
Francisco Borja	2008	Male	33	Yes (140/82 mmHg)	renal infarction	Yes	Right abdominal pain, vomiting	Normal	Not attempted
Francisco Borja	2008	Male	37	No	renal infarction	Not mentioned	left-sided abdominal pain	Normal	Not attempted
Jian-Ping Liu	2015	Male	43	Yes (158/85 mmHg)	Suprarenal abdominal aortic dissection	No	abdominal pain	Control (drugs)	Yes
Shu Wakino	2005	Famle	36	Yes (134/82 mmHg)	Renal infarction, subarachnoid hemorrhage	No	Left lower back pain, headache, loss of consciousness	Elevated after treatment (160/80)	No
Tadasuke Ando	2005	Male	31	No (130/60 mmHg)	renal infarction	Not mentioned	Sudden right lower back pain	Normal	Yes
Catherine M. Henry	2021	Male	39	Yes (130/71 mmHg)	renal infarction	7 packs/year (quit smoking for 4 months)	Right lower back pain, vomiting	Control (drugs)	Yes
Katia P. Souza	2022	Male	40	Yes (220/170 mmHg)	malignant hypertension	Not mentioned	abdominal pain	Control (drugs)	Yes
Mohammed Elhassan	2018	Male	36	No	renal infarction	No	Sudden right lower back pain after sex	Normal	Yes
Jitendra Parmar	2021	Male	41	Yes (>160 mmHg)	renal infarction	No	Bilateral low back pain	Control (drugs)	Yes
Cataldo Emanuela	2019	Male	48	Yes (140/100)	renal infarction	Not mentioned	Sudden severe back pain	Control (drugs)	Yes (spontaneous recirculation)
Gajapathiraju	2018	Famle	44	Yes (161/95 mmHg)	urinary tract infection	No	Right-sided abdominal pain, fever	Normal	No
Sophie Renaud	2012	Male	39	Yes (4/6 cases)	renal colic	1/6 cases	Dyspepsia, hematuria	Control (drugs)	Yes
T.L. Luk	2008	Male	42	No	renal ischemia	Not mentioned	Left-sided abdominal pain, vomiting	Normal	Yes
SP Stawicki	2006	Male	37	No	renal infarction	Not mentioned	Left-sided abdominal pain, hematuria	Normal	No
SP Stawicki	2006	Male	40	Yes(194/103 mmHg)	Bilateral renal infarcts	Not mentioned	Left-sided abdominal and back pain	Control (drugs)	Yes
SP Stawicki	2006	Male	61	Yes(190/95 mmHg)	renal infarction	Not mentioned	Left-sided abdominal pain, nausea, vomiting	Control (drugs)	No
M-J Hsieh	2012	Famle	51	Yes(187/81 mmHg)	renal infarction	Not mentioned	Epigastric pain, left-sided dystocia	Control (drugs)	No
J-F. Paul	2000	Male	57	Yes(190/100 mmHg)	renal infarction	Not mentioned	left upper abdominal pain	Control (drugs)	Yes
前鼻健志	2008	Famle	65	Yes	renal infarction	Not mentioned	Left upper abdominal pain, worsening renal function	Control (drugs)	Yes
沖貴士	2011	Famle	58	Yes(190/95 mmHg)	renal infarction	No	Right-sided abdominal pain, nausea	Normal	No
Joshua Bucher	2016	Male	55	Yes(150/89 mmHg)	Renal colic; aortic dissection	Not mentioned	Left-sided dystocia, nausea; retrosternal “crushing” chest pain with tearing sensation	Normal	Yes
Daniel Mudrick	2003	Male	47	Yes (severe hypertension)	Kidney stones (misdiagnosis)	No	Left dystocia, nausea, hematuria	Control (drugs)	Yes
Santhosh G. John	2010	Male	43	No(137/83 mmHg)	Acute pyelonephritis (misdiagnosis)	Yes	Lower left abdominal pain, nausea, vomiting, low-grade fever	Normal	No
Jamie Kanofsky	2007	Male	56	Yes(156/95 mmHg)	Kidney stones/pyelonephritis (misdiagnosis)	Yes	Right-sided dystocia, fever	Normal	Yes
P. Heim	1997	Male	48	Yes(200/100 mmHg)	Pharmacological hemolytic anemia (misdiagnosis)	Not mentioned	Right-sided dystocia (induced by cold medication)	Normal	No
Anam Haider	2017	Male	56	Yes(140/90 mmHg)	Pyelonephritis (misdiagnosis)	Not mentioned	Abdominal pain, vomiting, fever	Control (drugs)	No
Emanuele Casciani	2012	Male	47	Yes(145/95 mmHg)	Unspecified (emergency abdominal pain)	Not mentioned	right-sided pain	Normal	Yes
Jun-Yang Lou	2015	Male	32	Yes (not specified)	Pyelonephritis (misdiagnosis)	Not mentioned	Right-sided abdominal and back pain	Control (drugs)	No
Chami Im (case1)	2016	Male	50	Yes(173/100 mmHg)	Unspecified (abdominal pain)	Yes	Left lower abdominal pain	Control (drugs)	No
Chami Im (case2)	2016	Male	42	Yes(160/103 mmHg)	Unspecified (right dystocia)	Not mentioned	Right-sided dystocia, nausea, vomiting	Control (drugs)	No
Chami Im (case3)	2016	Male	35	Yes(161/95 mmHg)	Unspecified (left lower abdominal pain)	Not mentioned	Left lower abdominal pain	Normal	No
E. Guérin	2006	Male	47	Yes(150/100 mmHg)	Unspecified (emergency abdominal pain)	Not mentioned	Right abdominal pain → right dystocia	Control (drugs)	No
Tsung-Han Tsai	2010	Male	30	No(130/75 mmHg)	Kidney stones/pyelonephritis (misdiagnosis)	Not mentioned	Left dystocia, left lower abdominal pain	Normal	No
Siddharth Pandey	2018	Male	48	Yes(not specified)	Pyelonephritis (misdiagnosis)	Not mentioned	Right dystocia → abdominal pain → fever, necrotic lesions	Non-recovery (death)	No
Roberto G. Aru	2020	Male	28	Not mentioned	Chronic abdominal pain → rupture of mesenteric vessels	Not mentioned	Acute abdominal pain, hemorrhagic shock	Non-recovery (death)	No
R. M. Klein	1992	Male	33	Yes(190/110 mmHg)	Renal vein thrombosis (misdiagnosis)	Not mentioned	left-sided duress	Normal	Yes
Gerald S. Braun	2008	Male	31	No(130/75 mmHg)	Fitness exercise induced	Not mentioned	Sudden left dystocia while working out	Normal	Yes
Linda M. Reilly	1991	Male (8cases)	39.3	Yes(10/10)	Renal vascular hypertension	Not mentioned	Severe hypertension, neurological symptoms, hematuria, low back pain (8/10)	Control (drugs)	71.4%Yes
		Famle (2cases)							
C. Gun	2016	Male	39	Yes(170/93 mmHg)	suspected renal infarction	No	Left-sided abdominal pain, no hematuria	Normal	Not attempted
C. Gun	2016	Male	64	Yes(270/130 mmHg)	suspected renal infarction	Yes (20years)	Right-sided abdominal pain, nausea, vomiting	Normal	Yes
Tongli Yang	2024	Male	55	No(134/88 mmHg)	left renal space-occupying disease	Not mentioned	Left lumbar abdominal distension	Normal	Yes
Chunxu Liao	2020	Male	53	No	renal artery entrapment	No	Right-sided abdominal pain, low-grade fever	Normal	Not attempted
Qizhou He	2021	Male	49	No	entrapment of the left renal artery	Not mentioned	Swelling pain in the left lower back	Normal	Yes
Guqing Zhang	2011	Male	37	No(135/90 mmHg)	spontaneous renal artery entrapment	Not mentioned	Right-sided abdominal pain, fever	Normal	Not attempted
Qingyun Xu	2016	Male	39	Yes(165/98 mmHg)	Abdominal pain investigated	Not mentioned	Left-sided abdominal pain, nausea, vomiting	Control (drugs)	Not attempted
Zhongzhi Jia	2019	Male	64	Yes(135/85 mmHg)	Renal Artery Clamping Aneurysm	Not mentioned	Physical examination reveals a renal artery entrapment aneurysm	Normal	Yes
Moyang Wang	2009	Male	39	Yes(150/100 mmHg)	bilateral renal infarction	Yes (5years)	Low back pain, fever, elevated blood pressure	Normal	Yes
Hongying Yu	2013	Male	64	Yes (10-year history of hypertension)	entrapment of the left renal artery	Not mentioned	Difficulty in controlling high blood pressure	Normal	Yes
Lin Han	2018	Famle	36	Yes(199/132 mmHg)	renal artery entrapment	No	Hemoptysis, panic attacks, chest tightness	Normal	Yes
Guangqian Ding	2021	Male	49	No(115/70 mmHg)	Low back pain, microscopic hematuria	No	Sudden right-sided low back pain, nausea, profuse sweating	Normal	Not attempted
Kai Cui	2015	Male	46	No(107/68 mmHg)	right renal infarction	Not mentioned	Pain in the right lower back	not mentioned	Not attempted
Huimin Gao	2024	Male	76	Yes (up to 200/90 mmHg)	Double renal artery stenosis	Not mentioned	Fluctuating blood pressure, right lower extremity weakness	Normal	Yes

**Table 3:** Additional clinical data on patients.

Number	Fundamental disease	Post-operative complications	renal function	Mezzanine position	Diagnostic methods	therapeutic strategy	Survival rate at follow-up
1	Prostate cancer, premature ventricular contractions, Meniere’s disease	New-onset hypertension (postoperative)	Creatinine decreased from 270 to 190 μmol/L	Bilateral renal arteries	CT, CTA, US	Anticoagulation therapy → endovascular stent implantation	survival
2	not mentioned	Femoral artery puncture site hematoma	Decrease in creatinine	right renal artery	CTA, DSA	Balloon dilation stent graft implantation	survival
3	Hypertension for 5 years, kidney stones for 16 years	None	Stabilized renal function	Bilateral renal arteries	DSA, US, CT	Renal artery stent implantation Antihypertensive therapy	survival
4	None (relatively healthy)	None	Stabilized renal function	left renal artery	CTA	Conservative Treatment	survival
5	None (relatively healthy)	None	Stabilized renal function	Bilateral renal arteries, abdominal aorta	CTA, DSA, US	Conservative Treatment	survival
6	Hypertension for 12 years	Postoperative arteriovenous fistula, stent leakage	not mentioned	Vertebral artery, renal artery, iliac artery	DSA, CTA	Stent graft implantation Antiplatelet/anticoagulation	survival
7	None (relatively healthy)	None	Stabilized renal function	left renal artery	CT, CTA, US	Stent graft implantation Antiplatelet/anticoagulation	survival
8	None (relatively healthy)	Ischemic pancreatitis, acute kidney injury	Stabilized renal function	Abdominal trunk, superior mesenteric artery, bilateral renal arteries, right external iliac artery	CT, DSA, US	Anticoagulation of right renal artery angioplasty	survival
9	None (relatively healthy)	None	Stabilized renal function	left renal artery	CTA, DSA	Conservative Treatment	survival
10	None (relatively healthy)	None	Stabilized renal function	right renal artery	CT, DSA, US	Right nephrectomy	survival
11	None (relatively healthy)	Postoperative hematuria, total infarction of the left kidney	Total infarction of the left kidney with partial preservation of right kidney function	Bilateral renal arteries	CTA, DSA	Left renal artery stent implantation Thrombolysis	survival
12	None (relatively healthy)	None	Stabilized renal function	right renal artery	CTA, DSA, IVUS	Stent implantation	survival
13	Hypertension, COPD, sleep apnea	None	Stabilized renal function	right renal artery	DSA, IVUS, MRA	Stent implantation	survival
14	not mentioned	acute kidney injury	Mild chronic renal failure	left renal artery	CTA, DSA	Stent implantation	survival
15	Factor V Leiden mutation (weakly positive)	None	Stabilized renal function	Renal artery trunk and branches	CTA, DSA, MRI	Antiplatelet ACE inhibitors	survival
16	Tolosa-Hunt syndrome, corticosteroid therapy	None	Stabilized renal function	Celiac artery, splenic artery, SMA, right renal artery	CTA, DSA	Conservative treatment of SMA stents	survival
17	Fibromuscular dysplasia (1 case), atherosclerosis (1 case)	None	Stabilized renal function	renal artery	CTA, DSA	Conservative Treatment	survival
18	None (relatively healthy)	None	Stabilized renal function	right renal artery	CTA, DSA	Stent implantation	survival
19	Fibromuscular dysplasia	None	Stabilized renal function	renal artery	CTA, DSA	Conservative Treatment	survival
20	Degenerative lumbar scoliosis, osteophyte formation	None	Renal atrophy (infarcted area)	right renal artery	CTA, DSA	Conservative Treatment	survival
21	Fibromuscular dysplasia, mesangial hyperplasia	None	Stabilized renal function	left renal artery	DSA, Histopathology	Nephrectomy (left kidney)	survival
22	high blood pressure	renal necrosis	Deterioration of renal function (creatinine 3.5 mg/dL)	left renal artery	CTA, DSA	Nephrectomy after anticoagulation	survival
23	diabetes	None	Stabilized renal function	right renal artery	CT, MRI, DSA	Stent implantation	survival
24	Hypertension, diverticular disease	None	Stabilized renal function	Celiac artery, SMA, both renal arteries	CT, MRI, DSA	Anticoagulant SMA stents	survival
25	Ehlers-Danlos syndrome type IV	None	Partial preservation of renal function	right renal artery	MRI, DSA	Conservative Treatment	survival
26	None (relatively healthy)	None	Stabilized renal function	right renal artery	CT, DSA	Conservative Treatment	survival(3 months)
27	chronic hepatitis	None	Stabilized renal function	left renal artery	US, DSA	Stent implantation	survival(3 months)
28	None (relatively healthy)	None	Stabilized renal function	right renal artery	CT, DSA	Conservative Treatment	survival(17 days)
29	None (relatively healthy)	None	Stabilized renal function	left renal artery	CT, DSA	Conservative Treatment	None
30	Hypertension for 3 years	None	Stabilized renal function	Suprarenal abdominal aorta (involving celiac trunk)	CTA, DSA	Stent graft implantation	survival
31	Infertility treatment (hormone use)	Be in a coma	not mentioned	Both renal arteries, carotid artery, superior mesenteric artery, vertebral artery	CT, DSA	Anticoagulation, embolization	Death
32	None (relatively healthy)	None	Stabilized renal function	right renal artery	CT, MRI, DSA	Conservative Treatment	survival(4 months)
33	gout	None	Stabilized renal function	right renal artery	CT, US, DSA	Conservative Treatment	survival
34	Hypertension for 3 years	In-stent restenosis	Stabilized renal function	right renal artery	US, DSA, IVUS	Stent implantation Secondary balloon dilation	survival
35	Ulcerative colitis (in remission)	None	Stabilized renal function	right renal artery	CT, DSA	Conservative Treatment	survival
36	COVID-19 Hyperinflammatory state after pneumonia	Deep vein thrombosis of the lower limbs	Partial recovery	Renal artery trunk and branches	CT, DSA	Arterial thrombolysis balloon dilation	survival
37	Hypercholesterolemia, psoriasis, allergy asthma	None	Stabilized renal function	left renal artery	CTA, MRI	Conservative Treatment	survival
38	None (relatively healthy)	None	Stabilized renal function	right renal artery	CTA, DSA	Conservative Treatment	survival
39	Fibromuscular dysplasia (4/6 cases)	Not mentioned	Stabilized renal function	left renal artery	US, CTA, DSA	Anticoagulation, antihypertensive, some surgeries	survival
40	None (relatively healthy)	None	Stabilized renal function	left renal artery	CT, DSA	Angioplasty stents	survival
41	History of mitral valve prolapse, spontaneous pneumothorax	None	Stabilized renal function	left renal artery	MRA, DSA	Conservative Treatment	survival
42	fibromuscular dysplasia	None	Stabilized renal function	Bilateral renal arteries	CTA, DSA	Conservative Treatment	survival
43	None (relatively healthy)	None	Stabilized renal function	left renal artery	CT, DSA	Conservative Treatment	survival
44	Uterine fibroids, herniated discs	None	Stabilized renal function	left renal artery	CTA, DSA	Conservative Treatment	survival
45	None (relatively healthy)	None	Stabilized renal function	left renal artery	CTA	Stent implantation	survival
46	Hypertension, hyperlipidemia, hypothyroidism	None	Stabilized renal function	left renal artery	DSA, IVUS	Stent implantation	survival
47	Hypertension, atrial septal defect	None	Stabilization after right kidney removal	right renal artery	CT, CTA	nephrectomy	survival
48	Psoriasis, TMJ pain (hormone use)	Not mentioned	Stabilized renal function	left renal artery	CTA	Endovascular stenting Anticoagulation	survival
49	Hypertension (2 years history)	Not mentioned	Stabilized renal function	left renal artery	DSA	Coil embolization	survival
50	Positive antiphospholipid antibodies	Not mentioned	Stabilized renal function	left renal artery	CTA+Radionuclide Perfusion Imaging	Conservative Treatment	survival
51	None (relatively healthy)	Not mentioned	Stabilized renal function	right renal artery	CTA, DSA	Endovascular stenting	survival
52	Gilbert’s syndrome (Meulengracht’s disease)	Kidney function decreased to 17%	Functional compensation of the left kidney after right nephrectomy	right renal artery	US, CT, DSA	nephrectomy	survival
53	Benign prostatic hyperplasia, Helicobacter pylori gastritis	Not mentioned	Stabilized renal function	Bilateral renal arteries	CTA	Conservative Treatment	survival
54	None (relatively healthy)	Not mentioned	Stabilized renal function	right renal artery	MRA + DSA	Endovascular stenting	survival
55	History of cannabis use	Not mentioned	Stabilized renal function	right renal artery	CTA, IVUS	Conservative Treatment	survival
56	None (relatively healthy)	Not mentioned	Stabilized renal function	left renal artery	CTA	Conservative Treatment	survival
57	Hypertension (controlled)	Newly developed left renal artery entrapment	Stabilization of right renal infarction and disappearance of left renal thrombus	right renal artery	CTA	Conservative Treatment	survival
58	None (relatively healthy)	Fever, elevated white blood cells	Stabilized renal function	left renal artery	CTA	Conservative Treatment	survival
59	None (relatively healthy)	Not mentioned	Stabilized renal function	right renal artery	DSA + MRA	Conservative Treatment	survival
60	Positive antiphospholipid antibodies	Not mentioned	27% preserved left kidney function	left renal artery	CTA + DSA	Conservative Treatment	survival
61	not mentioned	Intestinal ischemia, multi-organ failure	Deterioration of renal function to death	right renal artery	CTA	Anticoagulation Surgery to remove a segment of the intestine	Death
62	Fibromuscular dysplasia (FMD)	multivessel complication	not mentioned	Multivessel (mesenteric, renal, iliac arteries)	CTA	Surgical resection of the bowel is supportive	Death
63	Unspecified (possible vasculopathy)	Not mentioned	10% left kidney function preserved	left renal artery	DSA	Conservative Treatment	survival
64	None (relatively healthy)	Not mentioned	Stabilized renal function	left renal artery	CTA	Conservative Treatment	survival
65	Fibromuscular dysplasia (7/10)	Nephrectomy after failed revascularization in 2 cases	Stabilized renal function	Renal artery trunk and branches	DSA, Histopathology	Revascularization (71.4%), nephrectomy (28.6%)	survival
66							
67	None (relatively healthy)	None	Stabilized renal function	left renal artery	CT, MRA	Conservative Treatment	survival
68	Diabetes, hypertension, atrial fibrillation, heart failure	None	Stabilized renal function	right renal artery	CT, CTA	Conservative Treatment	survival
69	None (relatively healthy)	In-stent leakage, endothelial proliferation, renal atrophy	Stabilized renal function	left renal artery	CTA, DSA	Interventional stent isolation and secondary repair	survival
70	None (relatively healthy)	None	Stabilized renal function	right renal artery	US, CTA	Conservative Treatment	survival
71	None (relatively healthy)	None	Stabilized renal function	left renal artery	CT, DSA	Coil embolization stent implantation	survival
72	None (relatively healthy)	None	Infarction of the middle and lower pole of the right kidney	right renal artery	CT, CTA	Conservative Treatment	survival
73	None (relatively healthy)	None	Stabilized renal function	left renal artery	CTA, DSA	Conservative Treatment	survival(3 months)
74	Left popliteal artery aneurysm	None	Stabilized renal function	right renal artery	CTA, DSA	Coil embolization Stent graft implantation	survival
75	None (relatively healthy)	None	Stabilized renal function	right renal artery	MRI, DSA	Stent implantation Thrombolytic therapy	survival(1 months)
76	History of abdominal trauma (splenectomy, left lower lobectomy)	None	not mentioned	left renal artery	DSA, IVUS	Renal artery stenting	survival
77	Myofiber dysplasia may	None	Stabilized renal function	left renal artery	CTA, DSA, IVUS	Stent implantation Antiplatelet therapy	survival
78	None (relatively healthy)	None	Stabilized renal function	right renal artery	CTA, DSA	Conservative Treatment	survival
79	None (relatively healthy)	Not mentioned	not mentioned	right renal artery	CTA, DSA	Conservative Treatment	survival
80	Coronary heart disease, diabetes mellitus, lower extremity atherosclerosis	None	not mentioned	left renal artery	CTA, DSA	Double renal artery stenting	survival

### 3.4. Diagnostic modalities

Computed tomography angiography (CTA) was the most utilized imaging modality (85.6%, 83/97), followed by digital subtraction angiography (DSA, 36.1%, 35/97), ultrasonography (14.4%, 14/97), and magnetic resonance imaging/angiography (MRI/MRA, 12.4%, 12/97). Multimodal imaging (≥2 modalities) was employed in 87.6% (85/97), most commonly CTA + DSA (19.6%, 19/97). Unilateral renal artery involvement occurred in 64.9% (63/97), with right (46.0%, 29/63) and left (54.0%, 34/63) arteries affected. Bilateral or multivessel dissection accounted for 35.1% (34/97).

### 3.5. Treatment strategies

Conservative management (36.1%, 35/97): Antihypertensive therapy (ACEI/ARB), anticoagulation (low-molecular-weight heparin), and serial imaging surveillance.

Endovascular intervention (57.7%, 56/97): Stent placement, thrombectomy, or angioplasty.

Surgical repair (6.2%, 6/97): Open surgical repair, nephrectomy, or vascular reconstruction.

### 3.6. Outcome measures

#### 3.6.1. Blood pressure recovery and renal function.

A total of 60 patients had complete blood pressure follow-up data (3 cases were excluded due to incomplete monitoring records: 1 lost to follow-up, 2 with irregular medication and blood pressure documentation). Overall, 96.7% (58/60) achieved blood pressure control (normotension without medication + controlled with medication). Subgroup analysis showed that the proportion of normotension without medication was significantly higher in the non-conservative group (54.1%, 20/37) than in the conservative group (30.0%, 6/20) (P < 0.05). Renal function stabilized or improved in 69.1% (67/97), while 30.9% (30/97) experienced deterioration or partial renal infarction.

#### 3.6.2. Revascularization and survival.

Revascularization rates were 37.1% (13/35) for conservative management (including 5.7% spontaneous recanalization) versus 71.0% (44/62) for interventional/surgical approaches (*P* < 0.01). Overall mortality was 3.1% (3/97), attributed to acute renal failure and multiorgan dysfunction.

#### 3.6.3. Complications, recurrence, and De Novo Dissection.

Postprocedural complications (limited to patients receiving endovascular or surgical intervention, n = 62) occurred in 29.0% (18/62), including endoleak (2 cases), acute kidney injury (7 cases), and access site hematoma (1 case). Recurrent or de novo dissection developed in 3 cases (3.1% of total patients): 2 recurrent cases in the endovascular intervention group (follow-up 6 and 12 months, respectively) and 1 de novo dissection in the conservative management group (follow-up 18 months, involving the contralateral renal artery).

## 4. Data analysis

### 4.1. Impact of treatment strategy on revascularization

A total of 97 SRAD patients from 73 studies were stratified by treatment strategy. The conservative management group (n = 35) achieved revascularization in 13 cases (37.1%) versus 22 failures (62.9%). In the non-conservative group (n = 62), revascularization succeeded in 44 cases (71.0%) versus 18 failures (29.0%). Risk ratio (RR) analysis demonstrated that conservative management was associated with a 0.52-fold lower likelihood of revascularization success compared to non-conservative strategies (RR = 0.52, 95% CI [0.29–0.74]). Odds ratio (OR) analysis further indicated that conservative management was associated with a 75.8% reduction in the odds of revascularization (OR=0.242, 95% CI [0.1–0.58]).

**Fig 3:**
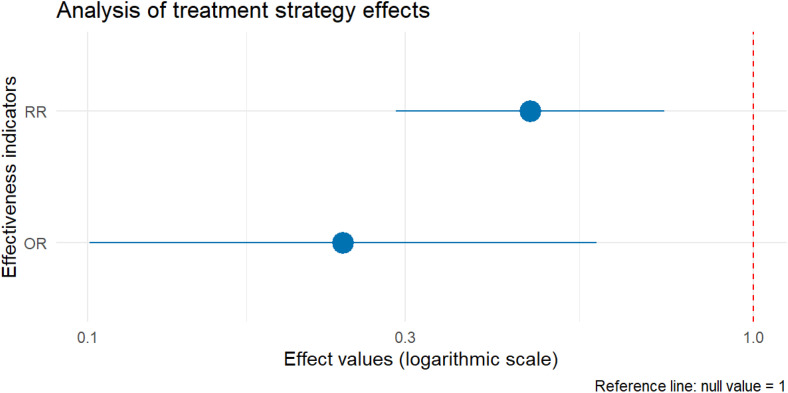
The effect size of treatment strategy on revascularization outcomes.

Chi-square testing revealed a statistically significant disparity in revascularization rates between groups (*χ*² = 9.2128, df = 1, *P* = 0.0024; *P* < 0.01). The effect size of treatment strategy on revascularization outcomes is illustrated in Fig 3.

### 4.2. Impact of treatment strategy on blood pressure recovery

Among 57 patients with complete subgroup blood pressure data (excluding 3 cases with incomplete records), blood pressure outcomes were categorized as normotension without medication or controlled with medication. In the conservative group (n = 20), 6 patients (30.0%) achieved normotension without medication versus 14 (70.0%) requiring medication. In the non-conservative group (n = 37), 20 (54.1%) achieved normotension without medication versus 17 (45.9%) controlled with medication. RR analysis suggested conservative management was associated with a 0.66-fold lower probability of blood pressure normalization without medication (RR = 0.52, 95% CI [0.42–1.03]). OR analysis indicated conservative strategies were associated with a 64.0% reduction in the odds of medication-free normalization (OR=0.36, 95% CI [0.11–1.16]).

**Fig 4:**
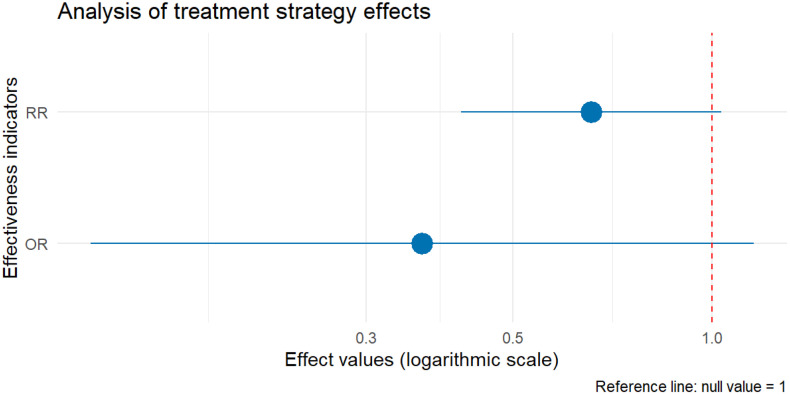
The effect size of treatment strategy on blood pressure outcomes.

Chi-square testing showed no statistically significant association between treatment strategy and overall blood pressure control rate (normotension without medication + controlled with medication) (χ² = 2.1359, df = 1, P = 0.1439; P > 0.05). The effect size of treatment strategy on blood pressure outcomes is detailed in Fig 4.

## 5. Discussion

Spontaneous renal artery dissection (SRAD), a rare non-traumatic vascular pathology, necessitates precise diagnostic and therapeutic decision-making to optimize patient outcomes. This study synthesizes data from 73 global publications to delineate SRAD’s epidemiological profile and clinical management. Our findings confirm prior reports of a middle-aged male predilection (mean age 46 years), likely due to higher hypertension and arteriosclerosis prevalence in this group. While acute-onset flank pain is the hallmark symptom, the observed 8.2% misdiagnosis rate—often misattributed to nephrolithiasis or pyelonephritis—underscores the imperative for heightened clinical vigilance in differential diagnosis [6]. Consistent with prior studies [1,3,5], our systematic review of 97 patients confirms a predilection for middle-aged males (mean age 46 years), with hypertension (61.9%) and acute-onset flank pain (74.2%) as the most common comorbidity and presenting symptom, respectively. Antopolsky et al. [1] reported similar demographic and clinical features in their 10-year ED study, while Yoon et al.[3] emphasized the role of hypertension and FMD in SRAD pathogenesis—findings that align with our comorbidity data (FMD in 16.5% of cases). Notably, the 8.2% misdiagnosis rate observed in our study (mostly as nephrolithiasis or pyelonephritis) echoes Jha et al.’s [5] systematic review (underdiagnosis rate >30%), underscoring the need for heightened clinical vigilance in differential diagnosis. Our study extends previous research by providing detailed subgroup analysis of blood pressure outcomes and recurrence risk, which were not fully addressed in Jha et al.’s [5] review.

Multimodal imaging protocols, particularly the integration of CTA and DSA, show complementary clinical value in SRAD diagnosis. CTA, with its superior spatial resolution and rapid acquisition, facilitates accurate delineation of dissection morphology (e.g., intimal tear location, false lumen extent), which is consistent with its role as the first-line imaging modality reported in 85.6% of our cases. DSA, as the gold standard for hemodynamic assessment, retains irreplaceable value in evaluating stenosis severity and collateral circulation—critical for guiding interventional therapy [1]. While our study did not perform head-to-head diagnostic efficacy comparisons, the high utilization rate of multimodal imaging (87.6%) in clinical practice reflects its practical utility in comprehensive evaluation. This aligns with clinical consensus that no single modality can address all diagnostic needs, and a stepwise algorithm combining CTA and DSA balances anatomical visualization and functional assessment.

Therapeutic strategy selection must be individualized based on symptom acuity, dissection progression, and renal functional reserve. Conservative management (antihypertensive/anticoagulant therapy) is suitable for hemodynamically stable patients without significant renal impairment [7,8], though its inferior revascularization efficacy (37.1% vs. 71.0% for interventional strategies) supports prioritizing endovascular intervention in symptomatic or radiographically progressive cases. It is important to note that these pooled estimates (RR, OR) are subject to inherent biases of case report data—including publication bias (successful interventional cases may be overrepresented) and selection bias (patients with milder symptoms may be more likely to undergo conservative management and underreported). Thus, the comparative efficacy of treatment strategies should be interpreted with caution, and these findings do not constitute definitive causal evidence. Proactive vascular repair may improve long-term outcomes in anatomically suitable lesions, but procedural risks (29.0% complication rate in the intervention group: endoleak, acute kidney injury, etc.) require careful risk-benefit assessment—particularly in elderly or high-comorbidity patients.

This analysis is constrained by several methodological factors: (1) reliance on retrospective case reports with inherent selection and reporting biases; (2) data heterogeneity, including incomplete documentation of smoking history and long-term follow-up; (3) the absence of randomized controlled trials (RCTs) to validate therapeutic disparities. (4) the inability to apply advanced meta-analytic techniques (e.g., meta-regression, sensitivity analysis) due to the inherent limitations of case report data—including lack of control groups, high clinical heterogeneity, and lack of prospective data collection.

These advanced methods are typically used to explore moderating variables or assess the robustness of pooled results, but their application here is methodologically unfeasible, further restricting causal inference from the pooled risk ratios (RR) and odds ratios (OR). It is important to note that these associations may be influenced by indication bias—patients with more severe symptoms or anatomical lesions are more likely to undergo interventional treatment—limiting causal inference.

Future multicenter prospective cohorts should establish standardized risk stratification frameworks, incorporating advanced imaging biomarkers and molecular profiling to guide individualized therapeutic algorithms.

## 6. Conclusion

The management of spontaneous renal artery dissection (SRAD) requires dynamic adjustment tailored to individual clinical characteristics and radiographic progression. While conservative therapy may serve as an initial strategy for hemodynamically stable patients, endovascular intervention demonstrates superior efficacy in achieving revascularization and should be prioritized in cases of symptomatic or anatomical deterioration. Clinicians must emphasize multimodal imaging integration to prevent diagnostic oversights and misclassification. Future research should focus on optimizing therapeutic timing, mitigating complication risks, and establishing evidence-based guidelines through randomized controlled trials (RCTs), ultimately improving long-term prognoses for SRAD patients.

## Supporting information

S1 File PRISMA flowchart. (DOCX)

S2 File PRISMA Checlist. (DOCX)

S3 File Supplyment. (ZIP)
