# Supplementary material for: Clinical characteristics, diagnostic modalities, and therapeutic strategies of spontaneous renal artery dissection: A systematic review and diagnostic analysis
Source: PLoS One. 2026 Jan 23;21(1):e0340766. doi: 10.1371/journal.pone.0340766 (PMC12829824; doi:10.1371/journal.pone.0340766)
Supplement: S1 File — (DOCX) [file pone.0340766.s001.docx]

**Identification of studies via databases and registers**

Records removed *before screening*:

Duplicate records removed (n =216 )

Records identified from*:

Databases (n = 539)

Registers (n =12)

**Identification**

Records screened

(n =335)

Records excluded**

(n =198)

**Screening**

Reports assessed for eligibility

(n = 137)

Reports excluded:

Reason 1 (n = 27)

Reason 2 (n = 37)

Reports of included studies

(n =73)

**Included**

* Literature Screening Process and Results *The databases searched and the number of documents detected are specified as follows：PubMed（n=141）、EMbase（n=4）、The Cochrane Library（n=43）、WOS（n=163）、CNKI（n=138）、WanFang Date（n=50）

**Automation tools not used
